# Application of Genome Sequencing from Blood to Diagnose Mitochondrial Diseases

**DOI:** 10.3390/genes12040607

**Published:** 2021-04-20

**Authors:** Rocio Rius, Alison G. Compton, Naomi L. Baker, AnneMarie E. Welch, David Coman, Maina P. Kava, Andre E. Minoche, Mark J. Cowley, David R. Thorburn, John Christodoulou

**Affiliations:** 1 Murdoch Children’s Research Institute, Melbourne, VIC 3052, Australia; rocio.rius@mcri.edu.au (R.R.); alison.compton@mcri.edu.au (A.G.C.); naomi.baker@vcgs.org.au (N.L.B.) annemarie.welch@mcri.edu.au (A.E.W.); david.thorburn@mcri.edu.au (D.R.T.); 2 Department of Paediatrics, University of Melbourne, Melbourne, VIC 3052, Australia; 3 Victorian Clinical Genetic Services, Melbourne, VIC 3052, Australia; 4 Department of Metabolic Medicine, Queensland Children’s Hospital, Brisbane, QLD 4101, Australia; David.Coman@health.qld.gov.au; 5 School of Clinical Medicine, University of Queensland, Brisbane, QLD 4072, Australia; 6 School of Medicine, Griffith University, Gold Coast, QLD 4222, Australia; 7 Department of Neurology, Perth Children’s Hospital, Perth, WA 6009, Australia; maina.kava@health.wa.gov.au; 8 Department of Metabolic Medicine and Rheumatology, Perth Children’s Hospital, Perth, WA 6009, Australia; 9 Kinghorn Centre for Clinical Genomics, Garvan Institute, University of New South Wales, Randwick, NSW 2010, Australia; andre@minoche.name; 10 Precision Medicine Theme, Children’s Cancer Institute, Kensington, NSW 2750, Australia; MCowley@ccia.org.au; 11 School of Women’s and Children’s Health, University of New South Wales, Randwick, NSW 2031, Australia

**Keywords:** mitochondria, heteroplasmy, genome sequencing, respiratory chain, mutation

## Abstract

Mitochondrial diseases can be caused by pathogenic variants in nuclear or mitochondrial DNA-encoded genes that often lead to multisystemic symptoms and can have any mode of inheritance. Using a single test, Genome Sequencing (GS) can effectively identify variants in both genomes, but it has not yet been universally used as a first-line approach to diagnosing mitochondrial diseases due to related costs and challenges in data analysis. In this article, we report three patients with mitochondrial disease molecularly diagnosed through GS performed on DNA extracted from blood to demonstrate different diagnostic advantages of this technology, including the detection of a low-level heteroplasmic pathogenic variant, an intragenic nuclear DNA deletion, and a large mtDNA deletion. Current technical improvements and cost reductions are likely to lead to an expanded routine diagnostic usage of GS and of the complementary “Omic” technologies in mitochondrial diseases.

## 1. Introduction

Mitochondrial diseases can be caused by pathogenic variants in more than 350 nuclear or mitochondrial DNA (mtDNA)-encoded genes that often lead to multisystemic and progressive symptoms [1].

In children, most primary mitochondrial diseases are caused by pathogenic variants in nuclear DNA (nDNA) (≈75%), while most adult-onset mitochondrial diseases result from mtDNA variants (≈75%) [2]. Since there are many mtDNA molecules within a cell, different levels of mutant mtDNA copies can coexist with wild-type mtDNA and the proportion can vary across tissues, which is a phenomenon known as heteroplasmy [3].

In recent years, the diagnostic approach of mitochondrial diseases has been revolutionized by massively parallel sequencing (MPS). In particular, Exome Sequencing (ES) has been increasingly adopted into clinical practice. This technology targets almost every coding region and flanking intronic nucleotides representing ≈2% of the genome. However, to analyze mtDNA, additional targeted mtDNA-MPS often needs to be performed. Alternatively, “off-target” reads from ES may be analyzed. A limitation of the ES “off-target” method is that detecting and determining low heteroplasmy variants can be challenging, as the sequencing depth is variable across the mtDNA [4]. Cohorts of patients with mitochondrial disease studied with ES have reported a molecular diagnostic yield between 35% and 70% [5,6,7,8,9,10,11,12,13,14]. The disparity between diagnostic yields likely reflects discrepancies in the stringency of inclusion criteria and thus heterogeneity between cohorts [2]. Nevertheless, achieving a diagnostic rate of more than 35% is a marked improvement on rates of 10–20% before the incorporation of MPS in mitochondrial disease diagnostics [1]. Genome sequencing (GS) can, in a single test, interrogate almost all the nDNA and mtDNA, with the advantage of efficiently detecting single nucleotide variants (SNV), short insertions and deletions, and structural variants (SV) in coding and non-coding regions of the nuclear genome, and can achieve a very high mtDNA read depth [15]. To date, only one cohort of patients with suspected mitochondrial disease studied through GS has been published, in which a definite molecular diagnosis was identified in 55% of the patients [15]. As the cost of GS declines and analysis becomes less complex, this technology is likely to be widely implemented in the near future for diagnosing mitochondrial diseases.

In this study, we aim to illustrate the utility of using GS in identifying different types of pathogenic variants in individuals with mitochondrial disease by providing case studies that emphasize the diagnostic advantages of this technology.

## 2. Materials and Methods

The cases presented in this series were selected from the Australian Genomics Health Alliance (AGHA) mitochondrial flagship genome sequencing (GS) arm [16]. All individuals had a history of suspected mitochondrial disease with a modified Nijmegen score > 4 [17].

Singleton PCR-free GS was performed in DNA extracted from blood and conducted by the Kinghorn Centre for Clinical Genomics at Garvan Institute of Medical Research (Sydney, Australia). Samples were sequenced on the Illumina HiSeq X Ten, with more than 75% of bases having Q30 base quality and mean coverage of > 30x. Reads were aligned to the b37d5 genome using Burrows–Wheeler Aligner (BWA) [18], sorted by coordinate using Novosort (Novocraft Technologies), realigned, and recalibrated using GATK to generate BAM files. BAM files were examined using the Integrated Genome Viewer 2.3 software (Broad Institute). Variants were called using GATK HaplotypeCaller (v3.4-46-gbc02625) [19], annotated using ENSEMBL’s Variant Effect Predictor [20] (v74), and converted to an SQLite database using Gemini [21] (v0.17.2). Databases were imported into SEAVE [22], which was used to perform phenotype-driven variant filtration and prioritization of the nDNA variants using a mitochondrial disease gene list (Mitochondrial disease v0.563, https://panelapp.agha.umccr.org/panels/203/, accessed on 27 December 2020), while mitochondrial SNV and indels were identified using *mity* [23]. SVs including copy number variants (CNVs) were investigated using ClinSV [24] (v1.0). In silico tools to analyze pathogenicity predictions included SIFT [25], Mutation Taster [26], PROVEAN [27], CADD [28], MitoTIP [29], PON-mt-tRNA [30], and HmtVar [31]. Classification of variants was based on the American College of Medical Genetics and Genomics (ACMG) guidelines [32].

Mitochondrial DNA sequencing was performed in skeletal muscle extracted DNA of P1 by the Victorian Clinical Genetics Services (Melbourne, Australia), DNA was extracted using the Macherey–Nagal DNA Extraction kit, according to the manufacturer’s instructions. The whole mitochondrial genome of 16.5 kb was amplified in a single long-range PCR, followed by library preparation using the Illumina Nextera DNA Flex kit (Illumina, (Illumina, San Diego, CA, USA). Sequencing was performed on a MiSeq (Illumina) using v2 chemistry, with a minimum coverage of 1000-fold. Data were processed using the on-board MiSeq Reporter for variant calling and a custom in-house analysis pipeline for annotation and indel calling as previously described [33].

Amplification of *MT-TL1* from urine extracted DNA of P1 was conducted by polymerase chain reaction (PCR) using primers designed to amplify 250 bp of mtDNA (Table S1), specificity for mtDNA and not mitochondrial NuMT was measured using DNA extracted from human rho0 cells [34] (cell line a kind gift from Dr Ian Trounce).

Total protein lysates were extracted from P2 fibroblasts for SDS-polyacrylamide gel electrophoresis and Western blotting using total Abcam OXPHOS human WB antibody cocktail (Abcam #ab11041) and Porin/VDCA1 (Abcam #ab14734) as previously described [35].

RNA was isolated from fibroblasts of P2 using the RNeasy Plus kit (Qiagen) following the manufacturer’s instructions. Reverse transcription was performed using SuperScript III First-Strand synthesis kit (Thermo Fisher Scientific, Carlsbad, CA, USA) following the manufacturer’s protocol. Amplification of complementary DNA (cDNA) was conducted by polymerase chain reaction (PCR) using three sets of primers designed to amplify exons 1–22 of *AARS2* (Table S1).

## 3. Results

### 3.1. Genome Sequencing Detects Low Levels of mtDNA Heteroplasmy in Blood

P1 is currently 49 years of age. He was well during childhood and early adult years but developed myoclonic epilepsy in his late 20s, which was triggered by a head injury. In his early 30s, he developed bilateral cataracts that required lensectomy. Over the ensuing 10 years, he developed profound sensorineural hearing loss, type 2 diabetes mellitus, non-alcoholic steatohepatitis, sensory peripheral neuropathy, ataxia, tremor, muscle cramping, and cognitive decline, such that he is no longer able to sustain work and struggles with independent living. A brain MRI scan demonstrated mild generalized cerebral atrophy.

**Figure 1:**
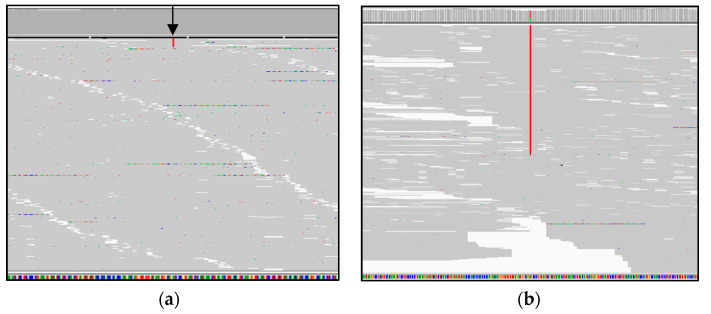

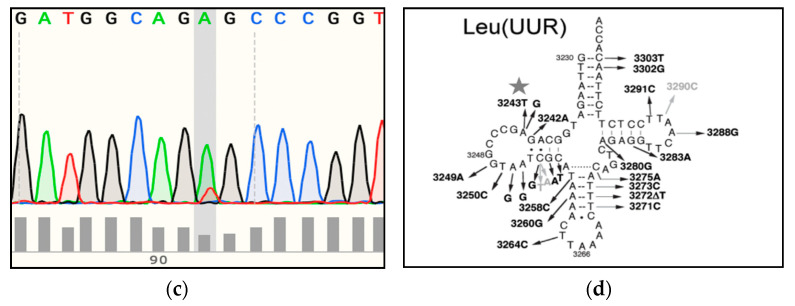
GS detects low levels of heteroplasmy in blood of P1. (**a**) Integrative Genomics Viewer (IGV) browser shows the identification of m.3243A>T in blood. The black arrow points to the variant (in red) present in 79 of 4235 reads aligned to the m.3243 position. (**b**) Partial electropherogram showing the heteroplasmic m.3243A>T variant detected in urine. (**c**) IGV browser shows the identification of m.3243A>T in muscle, the variant (in red) is present in 6108 of 8799 reads aligned to the m.3243 position. (**d**) Mamit-tRNA [38] schematic of the mt-tRNA^Leu(UUR)^ cloverleaf secondary structure showing in black positions of reported pathogenic variants. The m.3243A>T variant found in P1 is highlighted with a star and is located in the D-loop of the mt-tRNA^Leu(UUR)^.

Using GS in DNA extracted from blood, we identified a well characterized pathogenic mtDNA variant in the mitochondrial transfer RNA Leucine gene (*MT-TL1*) NC_012920.1(*MT-TL1*):m.3243A>T at 2% heteroplasmy in blood (Figure 1a). The variant was detectable in urine by Sanger sequencing suggesting heteroplasmic levels of at least 15% in urine sediment cells (Figure 1b) [36]. mtDNA-MPS was performed on DNA extracted from skeletal muscle, demonstrating a mutant load of 69% for the m.3243A>T variant (Figure 1c).

The variant is located within the D-loop of the mt-tRNA^Leu(UUR)^ (Figure 1d). The nucleotide (A) at this position has high evolutionary conservation (97.78%, MitoMaster) [37].

In silico software predicted this variant to be disease-causing. (Table 1). The variant was not detected in 56,423 individuals reported in the gnomAD v3.1 database [39].

This variant has been previously described as pathogenic in unrelated families with different mitochondrial phenotypes including mitochondrial encephalomyopathy with lactic acidosis and stroke-like episodes (MELAS MIM 540000), chronic progressive external ophthalmoplegia (CPEO), hearing loss with cataracts, and rhabdomyolysis. The variant was heteroplasmic in blood (5–46%) and muscle (30–81%) from the affected individuals [40,41,42]. Previous research using in vitro transcribed tRNA molecules showed that the m.3243A>T variant had a tRNA^Leu(UUR)^ aminoacylation efficiency only 1/300th that of the wild-type tRNA [43]. Furthermore, single fiber studies in patient muscle have shown higher amounts of mutant mtDNA in COX-deficient fibers compared with COX-positive fibers [40].

A skeletal muscle biopsy of patient P1 at age 48 showed variation in fiber size with scattered fibers demonstrating mild to moderate atrophy interspersed with preserved and relatively frequent hypertrophic fibers. Scattered granular fibers were present with a patchy increase in internal nuclei. Ragged red fibers were present using modified Gomori trichome staining. Scattered ragged blue fibers were present on succinyl-dehydrogenase (SDH) staining. There were increased numbers of cytochrome oxidase (COX)-deficient fibers with at least 25–30 per transverse section. Immunohistochemistry demonstrated patchy fiber type grouping indicative of chronic denervation.

The higher heteroplasmy level in an affected tissue compared with blood and urine, together with the genomic and muscle histochemistry, confirmed a diagnosis of mitochondrial disease in individual (P1).

This case illustrates how the high read depth of mtDNA in GS (>4000× depth in P1) enables the accurate detection of even low-level heteroplasmy variants [23]. For this reason, it is particularly useful when using blood as the source of DNA. For instance, it is well characterized that the mutant load in blood of the common m.3243A>G (the alternative nucleotide change to the variant identified in P1) decreases with age [44]. The m.3243A>G variant is the most common pathogenic mtDNA variant which at different heteroplasmy levels has been associated with MELAS, maternally inherited diabetes with or without deafness, myopathy, CPEO, and other mitochondrial cytopathies [3].

### 3.2. Genome Sequencing Detects an Intragenic Nuclear DNA Deletion

Patient P2 was born at term after an unremarkable pregnancy to non-consanguineous parents. He presented within an hour of birth with respiratory distress. He had persistent lactic acidosis in the newborn period with a peak lactate level of 16 mmol/L (normal range 0.7–2.0). Creatine kinase was 718 U/L on day 3 of life (normal range < 250 U/L). Echocardiogram was normal. Acylcarnitine profile, ammonia, full blood count, electrolytes, and liver function tests were normal.

The child continued to grow well until 4½ months of age when his length dropped from the 25th centile to the 10th centile. At nearly 5 months of age, he showed premature closure of the anterior fontanelle and slightly increased extensor tone in his legs with accompanying brisk deep tendon reflexes. The serum lactate level remained elevated at 4.5 mmol/L. He was started on a “mitochondrial cocktail” including thiamine, riboflavin, biotin, vitamin E, coenzyme Q10, and vitamin C at around 2 years of age.

At 3 years of age, he started having falls and showed delayed motor development and poor coordination. With intensive dietary therapy, his weight was maintained just above the third centile, and his height was 10–25th centile. His tone was reduced with both central and peripheral hypotonia. He had proximal myopathy and deep tendon reflexes were only just elicitable. He had hyperextensible joints and hypermobility. His speech was delayed and dysfluent. He had poor exercise tolerance and easy fatigability. Vision and hearing remained normal. There were no involuntary movements or seizures.

Repeat echocardiogram at 3 years of age was normal. EEG studies showed mild disorganization of the background but no epileptiform activity.

By 3½ years of age, he had global developmental delay, proximal myopathy, slowing of growth, and feeding difficulties. He is currently awaiting a gastrostomy tube insertion and continues to require regular occupational and physical therapy.

A skeletal muscle biopsy showed an increased number of fibers with subsarcolemmal aggregates of mitochondria on the SDH staining, and there were numerous COX-intermediate fibers. There were also multiple mini-cores noted on electron microscopy and mitochondrial pleomorphism. Skeletal muscle respiratory chain enzymes showed that complex IV activity was low relative to complex II and borderline low relative to citrate synthase.

**Figure 2:**
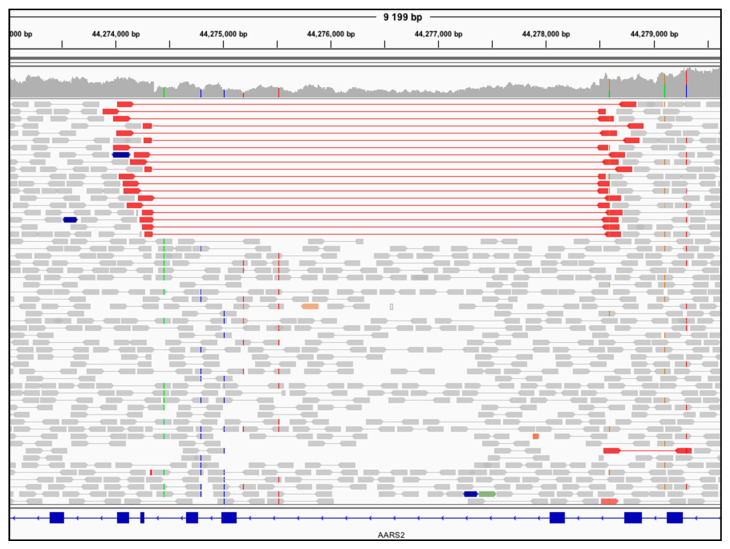
GS detects an intragenic nuclear DNA deletion in *AARS2* in P2. IGV coverage plot shows ≈50% fewer reads in the region spanning the deletion. The read pairs that are colored red are flanking the deletion.

The patient was studied through GS using DNA extracted from blood, which identified biallelic variants in the nuclear-encoded, mitochondrial alanyl-tRNA synthetase gene (*AARS2)*. A missense variant NM_020745.3(*AARS2*):c.2870C>T, NP_065796.1(AARS2):p.(Ser957Leu) and a 4.1 kb intragenic deletion spanning exons 5 through 7 (of 22) NC_000006.12:g.44274355_44278496del. The deletion was identified using ClinSV, which is a bioinformatic tool that integrates depth of coverage, discordant pairs, and split reads to detect SV from GS data [24] and by visual inspection of BAM files in the IGV browser [45] (Figure 2).

*AARS2* intragenic deletions affecting the same exons as P2 have been previously identified through ES and GS in two unrelated patients with AARS2-related mitochondrial disease [46,47]. However, the missense variant NM_020745.3(*AARS2*):c.2870C>T p.(Ser957Leu) has not been previously reported in other clinical cases. The serine at position 957 has very high evolutionary conservation, and the change to leucine represents a major amino acid change. It is located in the C-terminal globular subdomain of the AARS2 protein [48]. In silico software predicts this variant to be disease-causing (Table S2). It has been observed in the gnomAD population database at a frequency of 0.0000199 (5/251276) and has not been reported in a homozygous state.

PCR amplification of cDNA from fibroblasts identified three differently spliced transcripts resulting from the allele with the deletion. Two of these transcripts are predicted to induce a frameshift and a premature truncation NP_065796.2:p.(Glu251Serfs*27), NP_065796.2:p.(Gly98Profs*6) while the third transcript resulted in a shorter protein lacking 257 amino acids from the aminoacyl-tRNA synthetase IIc domain NP_065796.2:p (Glu146_Glu403del) (Figure S1). Using primers designed to amplify only the allele without the deletion (Primer set B, Table S1) showed the NM_020745.3(*AARS2*):c.2870C>T p.Ser957Leu variant appearing as hemizygous in the patient’s cDNA (Figure S2), and together, these results were indicative of compound heterozygosity. This was also confirmed by segregation testing using parental DNA.

**Figure 3:**
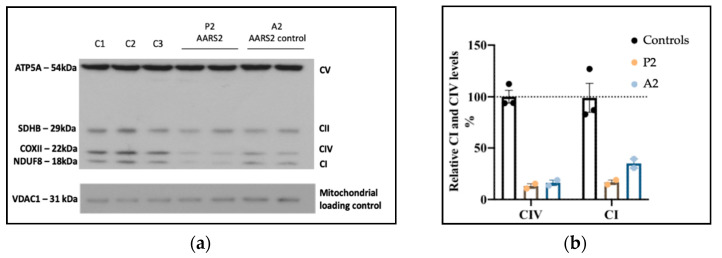
OXPHOS protein expression in patient P2 AARS2 and control fibroblasts. (**a**) Representative Western blot showing decreased protein levels of NDUFB8 (CI) and COXII (CIV) in patient P2 similar to patient A2 (positive control), who harbors likely pathogenic AARS2 variants c.1874G>A (p.Arg625His) and c.665C>T (p.Thr222Ile) in trans [49]; VDAC1 (porin) was used as a mitochondrial loading control. (**b**) Densitometry analysis suggests 85% lower levels of COXII (CIV) in both P2 and A2, and suggested 85% lower NDUFB8 (CI) in P2 and 65% lower in A2 relative to controls.

To investigate the functional impact of the *AARS2* variants on OXPHOS complexes, we performed Western blotting of fibroblasts using a cocktail of antibodies including NDUFB8, SDHB, UQCRC2, COXII, and ATP5A (OXPHOS human WB antibody cocktail, Abcam #ab11041). P2 fibroblast extracts showed decreased protein expression of OXPHOS COXII (Complex IV) and NDUFB8 (Complex I) subunits compared to control cells, while the levels of other complexes were normal (Figure 3).

Interestingly, decreased OXPHOS complex levels in fibroblasts in other patients with *AARS2* variants have not always been observed [50], and in some cases despite showing decreased AARS2 protein levels in fibroblasts, levels of OXPHOS proteins were only decreased in skeletal and cardiac muscle [46]. Variable reductions of different OXPHOS complexes and tissue specificity have been seen in other aminoacyl tRNA synthetases [51] and could be related to the type and location of the pathogenic variant. For instance, it has been proposed that patients with two “severe” or loss of function variants have an early-onset cardiomyopathy phenotype, due to increased requirements for mitochondrial protein synthesis in the developing heart, whereas patients with at least one variant leading to only a partial loss of protein levels or moderate reduction in aminoacylation activity could be associated with a predominant childhood-to-adulthood neurological phenotype [48]. Interestingly, distinct clinical presentations in the patients with the 4.1 kb deletion, but with different missense variants *in trans* have been observed. The deletion in combination with the p.(Arg580Trp) (located in the β-barrel domain and impacting the stability of the AARS2 protein) resulted in cardiomyopathy [46]. However, the deletion in combination with a p.(Arg199Cys) variant (located in the catalytic residue involved in ATP binding at the aminoacylation domain) [47,48] or the p.(Ser957Leu) variant identified in P2 (located in the C- terminal globular domain) was not associated with cardiomyopathy [47]. Further studies to experimentally measure the tRNA aminoacylation rate could contribute to our understanding of this potential association between genotype and phenotype.

Overall, using a GS approach allowed the prioritization and subsequently diagnostic confirmation of the *AARS2* variants in P2. This highlights the utility of GS and bioinformatic tools such as ClinSV to detect nuclear SVs.

### 3.3. Genome Sequencing Detects a Large Mitochondrial DNA Deletion

Patient P3 is a 14-year-old girl of unrelated parents. Her initial presentation was that of diabetic ketoacidosis at 4 years of age. Over the next 3 years, she developed sensorineural hearing loss, ptosis, CPEO, retinitis pigmentosa, short stature, and failure to thrive. She has required hormone replacement therapy for pubertal failure. Her cognition is normal and a brain MRI scan has been normal. Cardiology assessments have been normal including an echocardiogram, 12 lead electrocardiogram, and 24-h Holter monitoring.

**Figure 4:**
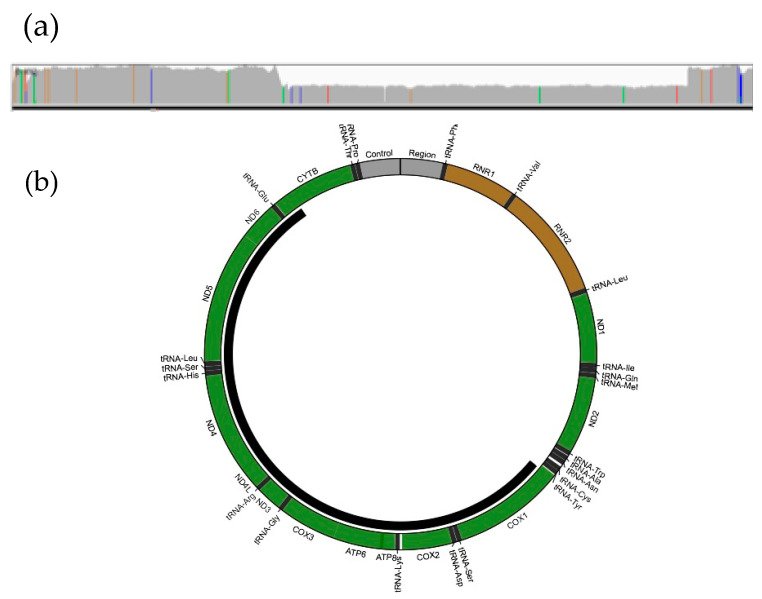
Deletion in the mtDNA in P3 identified by GS. (**a**) IGV coverage plot shows ≈50% fewer reads in the area spanning 9031bp of the mtDNA; (**b**) MitoBreak figure depicts the single large-scale heteroplasmic deletion spanning 23 mitochondrial genes (black bar).

A single large-scale pathogenic deletion in the mtDNA at a 50% level of heteroplasmy in blood was identified by GS (Figure 4a). This 9031 bp deletion encompasses 23 genes from *MT-CO1* to *MT-CYB* NC_012920.1:m.5965_14996del9031 (Figure 4b). Other single deletions in multiple individuals overlapping the deletion detected in P3 associated with mtDNA Deletion Syndromes have been previously reported in the MitoBreak database, and the phenotype in this patient is consistent with Kearns–Sayre syndrome (MIM 530000) [52].

The case discussed here demonstrates the capacity of GS to identify large heteroplasmic mtDNA deletions in blood. It is worth noting that mtDNA deletions in blood, similar to the case presented here, are often easier to detect in the pediatric population [53]. However, in adults, deletions in the mtDNA are often undetectable in blood and are preferentially found in post-mitotic tissues. Consequently, other tissues such as skeletal muscle may be a better source of DNA for testing in older individuals [54].

Change in heteroplasmy levels of single large-scale mtDNA deletions in mitotic tissues is exemplified by the natural history of some patients with Pearson syndrome (MIM 557000). Some infants with a single large-scale mtDNA deletion detectable in the blood often present with severe multisystemic disease with predominant hematological manifestations [55]. Interestingly, patients who survive beyond infancy can spontaneously recover from the hematological dysfunction, and the mtDNA deletion is no longer detectable in blood. However, in post-mitotic skeletal muscle, the deletion can accumulate with the phenotype evolving into Kearns–Sayre syndrome [53,56].

## 4. Discussion

Before the widespread adoption of MPS, the diagnostic approach to mitochondrial diseases often included invasive studies such as muscle biopsies as a first-line approach. However, more recently, many centers have evolved to a first-line MPS approach using DNA from blood or saliva in order to obviate the need for invasive testing, and so biopsies are needed only in selected cases such as when performing functional studies, testing mutant load in different tissues, or in adults with a suspected mtDNA deletion [57].

Using GS as a first-line molecular approach to diagnose patients with mitochondrial diseases has many advantages. One of the main benefits is the ability to efficiently analyze mtDNA as illustrated in P1 and P3. Unlike ES, GS does not require capture methods or “off-target” analysis, and as illustrated by P1, GS can achieve a high sequencing depth to detect low levels of heteroplasmy.

GS can also detect large mtDNA deletions as exemplified in P3. Moreover, PCR-free GS can accurately estimate an individual’s heteroplasmy levels using the relative read counts. In contrast, alternative MPS approaches for whole mtDNA sequencing often need a single long-range PCR step that may preferentially replicate shorter amplicons leading to amplification bias, and therefore, the heteroplasmy levels of a mtDNA deletion could be overestimated [58]. Therefore, additional quantitative approaches such as real-time PCR assays are needed to calculate heteroplasmy levels reliably [59]. Another potential problem with PCR-based approaches is that if SNVs are located at primer binding sites, the amplification efficiency may be affected [60].

As highlighted by P2 and P3, GS enables the detection of SVs in the nuclear and mitochondrial genomes, leveraging three independent lines of evidence: changes in read depth, split, and spanning reads. Copy number variants can also be identified from ES data, albeit only using changes in read depth [61]. For instance, an *AARS2* intragenic deletion also affecting the same exons as in P2 has been previously identified using ES [47]. However, GS is more reliable in detecting SV as the breakpoints can fall in non-coding regions. The data permit efficient interrogation of the distance between read pairs, split-reads, and they also have a more even sequencing coverage to use in read depth strategies [62]. Moreover, compared to ES, GS can achieve a more complete coverage of coding regions in clinically relevant genes, and therefore, GS could also be more effective in detecting SNV in the exome [63,64].

In contrast to ES, GS has the advantage that non-coding regions can be interrogated. However, the challenge remains in prioritizing and interpreting such variants, since the current understanding of the significance of variation in these regions is limited. Nevertheless, it is likely that bioinformatic analysis of non-coding regions will improve in the near future [65]. Therefore, as improved algorithmic approaches become available, GS provides the opportunity for continuing re-analysis of unresolved cases. Current practical strategies to prioritize and interpret non-coding regions such as deep intronic variants include targeted re-analysis of GS data in cases where a “single hit” in a coding region of a gene with a strong phenotype match is identified and/or performance of targeted RT-PCR [66], or using complementary “omic” approaches such as transcriptomics or RNA sequencing [67].

## 5. Conclusions

With reducing costs of sequencing, GS is a reliable option for first-tier diagnostic testing of patients with suspected mitochondrial disease. However, to date, no direct comparison between GS and ES have been published for mitochondrial disorders. Such studies are needed to provide guidance for implementation in health care settings, evaluation of cost-effectiveness, and guidance to overcome other potential barriers such as issues relating to computational and data storage infrastructure.

## Figures and Tables

**Table 1:** Pathogenicity predictions of the NC_012920.1(*MT-TL1*):m.3243A>T variant in P1.

In Silico Tool	Prediction (Score)	Reference
MitoTIP	Possibly Pathogenic (58.8%)	[29]
PON-mt-tRNA	Likely pathogenic (0.73)	[30]
HmtVar	Pathogenic (0.8)	[31]

## Data Availability

Not applicable.

## Supplementary Materials

The following are available online at https://www.mdpi.com/article/10.3390/genes12040607/s1, Table S1. Primers used for *MT-TL1* and *AARS2* analyses, Table S2. Pathogenicity predictions of the NM_020745.3(*AARS2*):c.2870C>T, p.Ser957Leu variant found in P2, Figure S1: *AARS2* deletion in P2 leads to three mutant transcripts, Figure S2. Sanger sequencing of allele specific *AARS2* PCR products in P2.
