# Supplementary material for: Application of Genome Sequencing from Blood to Diagnose Mitochondrial Diseases
Source: Genes (Basel). 2021 Apr 20;12(4):607. doi: 10.3390/genes12040607 (PMC8072654; doi:10.3390/genes12040607)
Supplement: Supplementary file 1 [file genes-12-00607-s001.zip › genes-1169594-SI.pdf]

Supplementary Material

Table S1. Primers used for *MT-TL1* and *AARS2* analyses

Target	Primer F Sequence	Primer R Sequence
MT-TL1	5′ CCTCCCTGTACGAAAGGACA 3′	5′ AGGAATGCCATTGCGATTAG 3′
A) *AARS2* exons 1-11	5′ ACTGCCCGGAGCTACGAT 3′	5′ CCAAGCGCATGGACATCAAG3 ′
B) *AARS2* exons 5-22	5′ ACACTGACCTCTTTCCCCG 3′	5′ GCCCATGTCTCCTTGTGTCA 3′
C) *AARS2* exons 20-22	5′ GGGACAGGCTGCAAAGAAAAC 3′	5′ TGCTGGGAGGGGCAAAACTA 3′

Table S2. Pathogenicity predictions of the NM_020745.3(*AARS2*):c.2870C>T, p.Ser957Leu variant found in P2

In silico tool	Prediction (Score)	Reference
SIFT	Deleterious (0.01)	[1]
Mutation Taster	Disease Causing (0.99)	[2]
PROVEAN	Damaging (-3.19)	[3]
CADD Scaled	1% most deleterious (25.9)	[4]

(a)

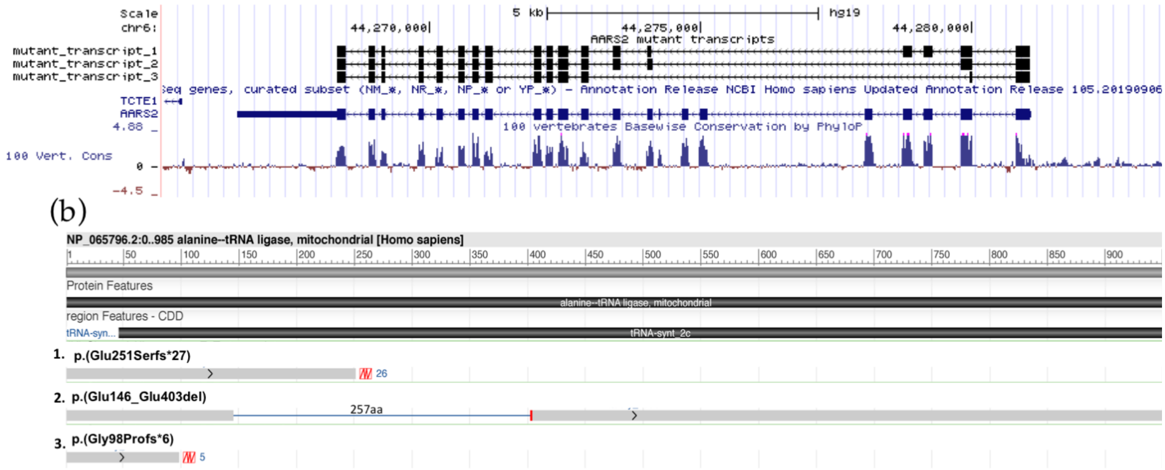

Figure S1. *AARS2* deletion in P2 leads to three mutant transcripts. (**a**) University of California, Santa Cruz (UCSC) Genome Browser screenshot showing the aligned cDNA sequences resulting from the allele with the deletion. Mutant transcript 1 resulted from a splicing event from exon 4 to exon 9, mutant transcript 2 from exon 2 to exon 9 and mutant transcript 3 from a cryptic splice site in exon 2 to exon 11 (**b**) At the protein level, the National Center for Biotechnology Information (NCBI) Sequence Viewer graphical display shows that mutant transcripts 1 and 3 are predicted to result in a frameshift and a truncation, and mutant transcript 2 to result in a shorter protein lacking 257 amino acids from the functional domain.

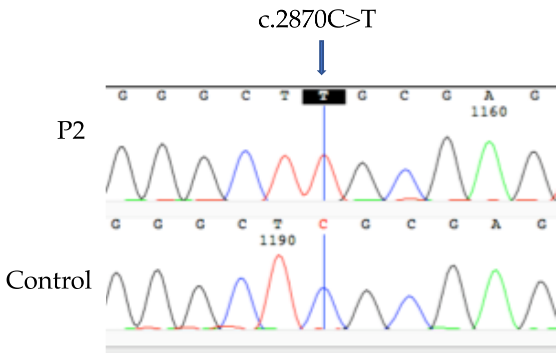

Figure S2. Sanger sequencing of allele specific *AARS2* PCR products in P2. The NM_020745.3(*AARS2*):c.2870C>T p.Ser957Leu variant appeared hemizygous in the cDNA from patient fibroblast when amplifying the allele without the deletion (Primer set B; exons 5-22).

1. Ng PC, Henikoff S, SIFT: Predicting amino acid changes that affect protein function. *Nucleic Acids Res* **2003,** 31 (13), 3812-4.
2. Schwarz JM, Rodelsperger C, Schuelke M, Seelow D, MutationTaster evaluates disease-causing potential of sequence alterations. *Nat Methods* **2010,** 7 (8), 575-6.
3. Choi Y, Sims GE, Murphy S, Miller JR, Chan AP, Predicting the functional effect of amino acid substitutions and indels. *PLoS One* **2012,** 7 (10), e46688.
4. Radivojac P, Clark WT, Oron TR, Schnoes AM, Wittkop T, Sokolov A, Graim K, Funk C, Verspoor K, Ben-Hur A, Pandey G, Yunes JM, Talwalkar AS, Repo S, Souza ML, Piovesan D, Casadio R, Wang Z, Cheng J, Fang H, Gough J, Koskinen P, Toronen P, Nokso-Koivisto J, Holm L, Cozzetto D, Buchan DW, Bryson K, Jones DT, Limaye B, Inamdar H, Datta A, Manjari SK, Joshi R, Chitale M, Kihara D, Lisewski AM, Erdin S, Venner E, Lichtarge O, Rentzsch R, Yang H, Romero AE, Bhat P, Paccanaro A, Hamp T, Kassner R, Seemayer S, Vicedo E, Schaefer C, Achten D, Auer F, Boehm A, Braun T, Hecht M, Heron M, Honigschmid P, Hopf TA, Kaufmann S, Kiening M, Krompass D, Landerer C, Mahlich Y, Roos M, Bjorne J, Salakoski T, Wong A, Shatkay H, Gatzmann F, Sommer I, Wass MN, Sternberg MJ, Skunca N, Supek F, Bosnjak M, Panov P, Dzeroski S, Smuc T, Kourmpetis YA, van Dijk AD, ter Braak CJ, Zhou Y, Gong Q, Dong X, Tian W, Falda M, Fontana P, Lavezzo E, Di Camillo B, Toppo S, Lan L, Djuric N, Guo Y, Vucetic S, Bairoch A, Linial M, Babbitt PC, Brenner SE, Orengo C, Rost B, Mooney SD, Friedberg I, A large-scale evaluation of computational protein function prediction. *Nat Methods* **2013,** 10 (3), 221-7.
